# Lip and Oral Cavity Cancer Incidence and Mortality Rates Associated with Smoking and Chewing Tobacco Use and the Human Development Index in 172 Countries Worldwide: An Ecological Study 2019–2020

**DOI:** 10.3390/healthcare11081063

**Published:** 2023-04-07

**Authors:** Antonio Hernández-Morales, Blanca Silvia González-López, Rogelio José Scougall-Vilchis, Josué Roberto Bermeo-Escalona, Ulises Velázquez-Enríquez, Rosalina Islas-Zarazúa, Sonia Márquez-Rodríguez, Taurino Amílcar Sosa-Velasco, Carlo Eduardo Medina-Solís, Gerardo Maupomé

**Affiliations:** 1 Doctoral Program in Health Sciences, School of Behavioral Sciences, Autonomous University of the State of Mexico, Toluca 50130, Mexico; ahernandezmo@uaemex.mx; 2 Faculty of Dentistry, Autonomous University of the State of Mexico, Toluca 50130, Mexico; jbermeoe764@profesor.uaemex.mx; 3 Advanced Studies and Research Center in Dentistry “Dr. Keisaburo Miyata” of Faculty of Dentistry, Autonomous University of the State of Mexico, Toluca 50130, Mexico; rscougallv@uaemex.mx (R.J.S.-V.); uvelazqueze@uaemex.mx (U.V.-E.); 4 Academic Area of Dentistry of Health Sciences Institute, Autonomous University of Hidalgo State, Pachuca 42160, Mexico; rosalina_islas8265@uaeh.edu.mx (R.I.-Z.); smarquez@uaeh.edu.mx (S.M.-R.); 5 School of Dentistry, Autonomous University “Benito Juarez” of Oaxaca, Oaxaca de Juárez 68120, Mexico; taurinoamilcar@hotmail.com; 6 Richard M. Fairbanks School of Public Health, Indiana University-Purdue University, Indianapolis, IN 46202, USA; gmaupome@iu.edu

**Keywords:** cancer, lip cancer, oral cancer, tobacco use, inequalities

## Abstract

Tobacco use is associated with diseases worldwide, including cancer. This is one of the major public health problems globally, causing more than 19 million new cases in 2020. Lip and oral cavity cancer (LOCC) is neoplastic growth in the tongue, gums, and lips. The objective of this ecological study was to quantify the strength of the association between incidence and mortality of LOCC, with tobacco use and with the Human Development Index (HDI). Incidence and mortality data on LOCC were obtained for 172 countries in 2020, from the Global Cancer Observatory (GLOBOCAN). The prevalence of tobacco smoking and chewing was obtained from reports conducted in 2019. The inequality in human development was estimated using the HDI from the United Nations Development Program, Human Development Report (2019). Statistically significant correlations were observed between the incidence of LOCC and tobacco smoking and chewing prevalence, except for negative correlations between the prevalence of tobacco smoking LOCC mortality in women, just as in the case of the HDI. No statistically significant differences were found between the prevalence of tobacco chewing only and the incidence of LOCC overall and by sex. A higher LOCC incidence overall and by sex was associated with higher HDI. In conclusion, the present study found positive correlations for various HDI socioeconomic indicators and tobacco use with the incidence and mortality of LOCC, but also a few inverse correlations.

## 1. Introduction

According to the Global Burden of Disease, it is shown that oral conditions continue to be a major challenge in terms of population health. Worldwide, there were approximately 3.5 billion cases with oral conditions, where 2.3 billion had untreated caries in permanent teeth, 796 million had severe periodontitis, 532 million had total tooth loss, and 139 million had other oral conditions [1]. Oral diseases are among the most prevalent diseases globally and have serious health and economic burdens, greatly reducing the quality of life for those affected. The most prevalent and consequential oral diseases globally are dental caries, periodontal disease, tooth loss, and cancers of the lips and oral cavity [2]. These data show that oral diseases and their consequences are public health problems that the world’s health systems must face [3]. Oral diseases, while largely preventable with simple actions [4,5,6], pose a major health burden for many countries and affect people throughout their lifetime, causing pain, discomfort, disfigurement, and even death [7].

Cancer is one of the major public health problems worldwide (incidence 19,292,789 cases and mortality 9,958,133 cases in 2020), and it is the disease with the highest morbidity and mortality of our time [8,9], anticipated to be the cause of death for 13.2 million people by 2030 [10]. Oral cancer includes cancers of the lip, other parts of the mouth and the oropharynx, and combined ranks as the 13th most common cancer worldwide. Lip and oral cavity cancer (LOCC) is the most common malignant neoplasm of the head and neck; it is a proliferation of malignant epithelial cells that frequently involves the lower lip, the anterior two-thirds of the tongue, the floor of the mouth, and the alveolar process of the maxilla and the mandible [11,12]. The global incidence of cancers of the lip and oral cavity is estimated to be 377,713 new cases and 177,757 deaths in 2020. Oral cancer is more common in men and in older people (between the ages of 50 and 60), is more deadly in men compared to women, and varies strongly according to socioeconomic circumstances [7,8,11,12].

Worldwide, tobacco use is responsible for many diseases, including cancer; its use is reported in a large percentage of oral cavity cancer cases, constituting the main risk factor, in addition to alcohol consumption, betel quid, and HPV infection [13]. Tobacco can be smoked, which directly enters the respiratory tract and spreads its substances systemically, or smokeless, where the substances come into direct contact with the oral mucosa [14,15]. The different forms of consumption largely depend on the region of the world; in Western countries, the smoked form of tobacco is the most common, while in Asian countries, the smokeless form is the most common [16]. In addition, alcohol consumption in combination with tobacco use significantly increases the risk of developing cancer due to their synergistic effect. High-risk human papillomavirus subtypes HPV16 and HPV18, chronic trauma, immunodeficiency, and nutritional deficiencies play an important role in the development of oral cancer. Exposure to ultraviolet radiation induces changes in skin tissues that are predisposed to lip cancer formation [17,18,19]. In addition to the abovementioned risk factors, there are various socioeconomic factors, related to individual or contextual inequalities, that may have a negative impact on the different types of cancer; inequality in human development is a key socioeconomic determinant of cancer, as is the case with LOCC.

In the present report we hypothesized that the worse the Human Development Index score (and its components), the higher the incidence and mortality for LOCC. We conducted separate and joint analyses incorporating smoking and chewing tobacco use and the HDI in 172 countries.

## 2. Materials and Methods

Because secondary data from public databases were used in the present ecological study, approval by an ethics and research committee was not required for this specific study, as approved and undertaken by the Academic Area of Dentistry at the Autonomous University of Hidalgo State (UAEH-ICSA-ODO-063).

### 2.1. Variables

The International Agency for Research on Cancer is responsible for providing global estimates on the burden of cancer worldwide, the results of which are published through GLOBOCAN, and is responsible for updating the estimates on the incidence and mortality for each type of cancer for 2020 [20]. The incidence and mortality data provided by GLOBOCAN serve to evaluate the performance of cancer prevention and treatment strategies implemented by each country [21]. The data were obtained from the Global Cancer Observatory (GLOBOCAN) database, (http://globocan.iarc.fr/today/ (accessed on 10 July 2022)). It includes information on age-standardized incidence and mortality rates for lip and oral cavity cancer for 172 countries for 2020, reported per 100,000 inhabitants aged between 10–85 years old (it is a publicly accessible database).

The prevalence of tobacco smoking and chewing was obtained from reports by Reitsma et al. [22] and Kendrick et al. [23], respectively. The overall average prevalence of tobacco smoking and chewing, as well as its prevalence by sex (tobacco smoking), was reported per 100,000 inhabitants, while tobacco chewing was described in percentages.

The Human Development Index (HDI) was created with the aim of establishing a real scale for personal growth in a given country, focusing on three dimensions: education, standard of living, and health [24,25]; it is a summary measure and classifies countries as very high, high, medium, and low HDI [26]. According to this index, countries with a high HDI score present faster development than those with a medium or low index. However, the HDI does not fully and accurately reflect inequality, poverty, and human security for each country; thus, the analysis of such indicators must be performed additionally [26,27]. The HDI was collected from the United Nations Development Program, Human Development Report (https://hdr.undp.org/data-center/human-development-index#/indicies/HDI (accessed on 10 July 2022)); the indicators for life expectancy, expected years of schooling, and average years of schooling were obtained for 172 countries in 2019.

### 2.2. Analysis

The association of LOCC incidence and mortality with the prevalence of tobacco smoking and chewing and the HDI was determined using the Spearman’s correlation test (*p* ≤ 0.05).

## 3. Results

### 3.1. Global Epidemiology of LOCC

Overall, the LOCC global incidence was 377,713 cases in 2020, with most cases occurring in men (264,211 cases). In terms of mortality, 177,757 deaths were reported; most deaths were observed in males, with a total of 125,022.

Table 1 shows (Table S1) the incidence rates of LOCC for 172 countries. The countries with the lowest number of cases were São Tomé and Príncipe (equatorial Atlantic), Belize, and Nicaragua (Central America), while the highest incidence was detected in Asian countries, with Papua New Guinea (Melanesia), Pakistan (south Asia), and India (Hindustan) being the countries with the highest incidence of LOCC. The incidence by sex detected Papua New Guinea as the country with the highest incidence in men, followed by Sri Lanka, and India, while in women, the highest number of new cases were found in Papua New Guinea, Pakistan, and Bangladesh.

The LOCC mortality rates are shown in Table 2 (Table S2). The countries with the highest incidence were Papua New Guinea, Pakistan, and Bangladesh, while the countries with the lowest mortality rates were São Tomé and Príncipe, the Maldives, and Belize. The countries with the highest mortality rates in men were Papua New Guinea, Pakistan, and India, while the countries with the highest mortality rates in women were Papua New Guinea, Pakistan, and Bangladesh.

### 3.2. Global Prevalence of Tobacco Smoking and Chewing

Tobacco smoking use in men showed higher prevalence in Timor-Leste, Micronesia, and Indonesia. In women, Bosnia and Herzegovina showed the highest prevalence of tobacco smoking, followed by Serbia, and Micronesia.

With regard to tobacco chewing in men, Nepal, Bhutan, and India were the countries with the highest prevalence, while in Bangladesh chewing tobacco use in women showed a higher prevalence, even more so than in men. In Bhutan and Cambodia, chewing tobacco use in women was also prevalent.

### 3.3. Human Development Index

The countries with the highest Human Development Index score were Norway, Ireland, and Switzerland, while those with the lowest were Niger, the Central African Republic, and Chad.

Japan was the country with the highest life expectancy (84.6 years), and the country with the lowest was the Central African Republic (53.3 years). Australia was the country with the highest expected years of schooling (22 years), while the lowest was Eritrea (5 years). In terms of the average years of schooling, Germany was the best rated (14.2 years) and the worst was Burkina Faso (1.6 years).

### 3.4. Correlation of LOCC Incidence and Mortality with the Prevalence of Tobacco Smoking and Chewing

A positive correlation was observed between the incidence of LOCC and the average level of tobacco smoking (*p* < 0.001, *r* = 0.394), as well as between the incidence of LOCC in men and the prevalence of tobacco smoking (*p* < 0.001, *r* = 0.286). A positive correlation was found between the incidence of LOCC in women and the prevalence of tobacco smoking in women (*p* < 0.001, *r* = 0.253).

No statistically significant differences were found between the prevalence of tobacco chewing and the incidence of LOCC overall and by sex.

The analysis between LOCC mortality and the average level of tobacco smoking revealed a positive correlation (*p* = 0.005, *r* = 0.214), similar to the correlation between the prevalence of tobacco smoking in men and LOCC mortality (*p* = 0.001, *r* = 0.249). The opposite occurred with the prevalence of tobacco smoking in women and LOCC mortality in women, where a negative correlation between these variables was obtained (*p* = 0.003, *r* = −0.227).

The analysis between LOCC mortality and the average level of tobacco smoking revealed a positive correlation (*p* = 0.005, *r* = 0.214), similar to the correlation between the prevalence of tobacco smoking in men and LOCC mortality (*p* = 0.001, *r* = 0.249). The opposite occurred with the prevalence of tobacco smoking in women and LOCC mortality in women (*p* = 0.001, *r* = 0.249). 001, *r* = 0.249), where a negative correlation between these variables was obtained (*p* = 0.003, *r* = −0.227).

Tobacco chewing showed a positive correlation between the average level of tobacco chewing and LOCC mortality (*p* = 0.030, *r* = 0.166), and between the prevalence of tobacco chewing in women and mortality in women (*p* < 0.001, *r* = 0.322) (Table 3).

### 3.5. Correlation of LOCC Incidence and Mortality with the Human Development Index (HDI)

The HDI showed a positive correlation with the overall LOCC incidence (*p* < 0.001, *r* = 379), as well as with the incidence in men (*p* < 0.001, *r* = 0.374) and in women (*p* < 0.001, *r* = 0.268). Life expectancy presented a positive correlation with the overall LOCC incidence (*p* < 0.001, *r* = 0.290), in the same way as the incidence in men (*p* < 0.001, *r* = 0.273) and in women (*p* = 0.002, *r* = 0.232). The expected years of schooling correlated with the overall LOCC incidence (*p* < 0.001, *r* = 0.352), in the same way as the incidence in men (*p* < 0.001, *r* = 0.343) and in women (*p* = 0.001, *r* = 0.203). Finally, the average years of schooling showed a correlation with the overall LOCC incidence (*p* < 0.001, *r* = 0.387), and also with the incidence in men (*p* < 0.001, *r* = 0.417) and in women (*p* = 0.008, *r* = 0.203).

In contrast, the LOCC mortality presented a negative correlation with life expectancy (*p* = 0.005, *r* = −0.213). Something similar occurs with LOCC mortality in women, which showed a negative correlation with the HDI (*p* < 0.001, *r* = −0.319), life expectancy (*p* < 0.001, *r* = −0.356), expected years of schooling (*p* < 0.001, *r* = −0.423), and average years of schooling (*p* < 0.001, *r* = −0.307) (Table 4).

## 4. Discussion

We analyzed the incidence and mortality rates of LOCC in 172 countries, and their correlation with the prevalence of tobacco smoking and chewing, and with the components of the Human Development Index (HDI). The findings showed that the highest incidence of LOCC, overall and by sex, was found in countries in Oceania and Asia, countries with a history of high tobacco use in its various forms. Similar results have been reported previously, southeast Asian countries have a higher incidence of LOCC and a correlation with the geographical distribution of betel nut [28], which is considered a major risk factor for the development of potentially malignant lesions and oral cancers [29]. It is necessary, however, to undertake future studies to further delineate such phenomena.

LOCC mortality has increased in the last 30 years, mainly in western Pacific countries. An exception are African countries, which reported reduced mortality rates [30], results that are consistent with ours.

Tobacco use, whether smoked or chewed, is still considered an epidemic and, despite a reduction in its consumption, it should be treated as a pandemic. About 1.3 billion people use tobacco and it has been responsible for more than 6 million deaths. The World Health Organization (WHO) predicts that by the year 2030, this figure will increase to 8.3 million deaths [31,32].

As expected, high smoking tobacco use is associated with high incidence rates of LOCC, and these results are consistent with previous studies, noting that both the amount and time of exposure play an important role [33]. Likewise, Al-Zalabani [34] reported that tobacco smoking was responsible for most cancer cases in countries such as Qatar, Bahrain, and the United Arab Emirates, mainly affecting women. In our study, we identified the relationship between the prevalence of smoking tobacco and an incidence increase in LOCC in women and men in a similar way. It remains important to implement programs that encourage the disuse of this product to prevent oral diseases [29].

Although the prevalence of chewing tobacco and LOCC incidence was not significantly correlated, the WHO stated that this product continues to be highly prevalent in south Asian countries, in both men and women, and is associated with LOCC [35,36]. In terms of the differences in associations pertaining to smoked and chewed tobacco, we propose that even though non-chewed tobacco may have higher nitrosamine content (a major carcinogen), it lacks the addition of heat derived from smoking tobacco. This could be a factor of yet undetermined importance. Smoked tobacco may have polycyclic aromatic hydrocarbons like pyrene, which is highly carcinogenic [37].

Through analyzing the results for LOCC mortality, it was seen that when the prevalence of tobacco smoking increased, the mortality from LOCC also increased, particularly in men. Its prevalence is higher in Asian countries. In women, however, the prevalence is higher in European countries. These results are consistent with the study by Sao Jose et al., which indicated that although there was a decrease in the mortality from lung, trachea, and bronchus cancer, LOCC showed a 30% increase in deaths [32]. For women in the present study, we observed that when the prevalence of tobacco smoking increased, there was a reduction in the mortality. Further studies are needed to analyze the relevant data in more detail to better characterize such a paradoxical trend.

When tobacco chewing increased, there was an increase in LOCC mortality among women, but no significant differences were found in men. These results are very similar to those found by Inoue-Choi et al. [38], where they mentioned that the exclusive use of smokeless tobacco had a strong association with oral cavity cancer mortality. The fact that we found a significant difference in women may be supported by the study by Dandona et al. [39], in that the use of smokeless tobacco by women is reported to be equal to or even higher than that by men in several countries, especially in south Asian countries.

When comparing the Human Development Index with LOCC incidence, it was found that the higher the HDI, the higher the LOCC incidence. This could be associated with a higher income and better access to health services, so that LOCC can be more accurately diagnosed. Kurivilla et al. reported similar results and indicated that developed countries had higher incidence rates; this feature may be associated with greater capabilities of their health systems to attain earlier cancer diagnoses [40]. Freire et al. concurred with such explanation [41]. Xie et al. proposed that in more developed countries, not only does the consumption of tobacco and alcohol increase, but life expectancy is also higher. This demographic feature allows for a longer time window for cancer to occur [42]. In contrast, a negative correlation between the HDI and LOCC was found for LOCC mortality, especially in women. Considering that mortality is aligned with incidence, but the situation is complicated by clinical care issues of its own, more precarious access to healthcare services in less developed countries likely leads to later diagnosis and higher mortality rates [43].

The study we conducted has some limitations. The first is that in an ecological study, the results cannot be extrapolated or linked to individual cases in particular. The second is that some other risk factors were not included in the study, such as alcoholism, HPV infection, sun exposure, etc. A broader study is needed to incorporate information about other risk factors. Finally, there is the limitation that in tobacco chewing, the modality of use may have varied: it could be the consumption of betel quid with tobacco, areca nut, etc., which depends on the country/region.

## 5. Conclusions

The incidence and mortality of LOCC are associated with the prevalence of tobacco smoking, as well as with the HDI. Such confirmatory findings emphasize the need to better control the commercial determinants of tobacco use, and to improve intervention treatment, health promotion, health policy, and create more awareness to reduce tobacco use in all its presentations. Such increased attention should be especially important in countries with a low HDI.

## Figures and Tables

**Table 1:** Incidence of LOCC, 10 countries with the highest and 10 with the lowest incidence rates.

Country	Rank	Incidence per 100,000	Average Tobacco Smoking per Day	Average Tobacco Chewing %	HDI Value	Life Expectancy Years	Expected Years of Schooling	Average Years of Schooling
Papua New Guinea	1	27.1	29.3	5.79	0.555	64.5	10.2	4.7
Pakistan	2	13.0	14.23	9.47	0.557	67.3	8.3	5.2
India	3	12.5	13.05	18.75	0.645	69.7	12.2	6.5
Sri Lanka	4	12.4	15.95	9.36	0.782	77.0	14.1	10.6
Bangladesh	5	12.2	23.51	23.68	0.632	72.6	11.6	6.2
Namibia	6	8.4	17.05	0.87	0.646	63.7	12.6	7.0
Australia	7	8.3	15.4	0.41	0.944	83.4	22	12.7
Hungary	8	8.1	30.35	0.21	0.854	76.9	15.2	12.0
Slovakia	9	7.9	27.1	0.22	0.860	77.5	14.5	12.7
Cape Verde	10	7.7	6.19	1.54	0.665	73.0	12.7	6.3
Guinea	163	1.2	15.61	0.83	0.477	61.6	9.4	2.8
Panama	164	1.2	8.48	0.37	0.815	78.5	12.9	10.2
Jamaica	165	0.96	13.46	0.46	0.734	74.5	13.1	9.7
Algeria	166	0.86	17.22	4.01	0.748	76.9	14.6	8.0
Bolivia	167	0.79	12.75	0.75	0.718	71.5	14.2	9.0
Brunei Darussalam	168	0.75	16.94	0.87	0.838	75.9	14.3	9.1
Congo	169	0.67	11.25	0.66	0.574	64.6	11.7	6.5
Nicaragua	170	0.64	13.48	0.65	0.660	74.5	12.3	6.9
Belize	171	0	13.31	0.52	0.716	74.6	13.1	9.9
São Tomé and Príncipe	172	0	4.75	0.47	0.625	70.4	12.7	6.4

**Table 2:** LOCC mortality, 10 countries with the highest and 10 with the lowest incidence rates.

Country	Rank	Mortality per 100,000	Average Tobacco Smoking per Day	Average Tobacco Chewing %	HDI Value	Life Expectancy Years	Expected Years of Schooling	Average Years of Schooling
Papua New Guinea	1	10.6	29.3	5.79	0.555	64.5	10.2	4.7
Pakistan	2	8.2	14.23	9.47	0.557	67.3	8.3	5.2
Bangladesh	3	7.2	23.51	23.68	0.632	72.6	11.6	6.2
India	4	7.0	13.05	18.75	0.645	69.7	12.2	6.5
Sri Lanka	5	5.8	15.95	9.36	0.782	77.0	14.1	10.6
Namibia	6	4.7	17.05	0.87	0.646	63.7	12.6	7.0
Afghanistan	7	4.2	9.83	5.34	0.511	64.8	10.2	3.9
Hungary	8	4.1	30.35	0.21	0.854	76.9	15.2	12.0
Belarus	9	4.0	36.9	0.25	0.823	74.8	15.4	12.3
Poland	10	3.6	28.1	0.32	0.880	78.7	16.3	12.5
Israel	163	0.47	20.65	0.19	0.919	83.0	16.2	13.0
Jamaica	164	0.46	13.46	0.46	0.734	74.5	13.1	9.7
Congo	165	0.45	11.25	0.66	0.574	64.6	11.7	6.5
Nicaragua	166	0.43	13.48	0.65	0.660	74.5	12.3	6.9
Brunei Darussalam	167	0.41	16.94	0.87	0.838	75.9	14.3	9.1
Bolivia	168	0.38	12.75	0.75	0.718	71.5	14.2	9.0
Algeria	169	0.37	17.22	4.01	0.748	76.9	14.6	8.0
Belize	170	0	13.31	0.52	0.716	74.6	13.1	9.9
Maldives	171	0	26.92	4.62	0.740	78.9	12.2	7.0
São Tomé and Príncipe	172	0	4.75	0.47	0.625	70.4	12.7	6.4

**Table 3:** Correlation between the prevalence of tobacco smoking and the incidence and mortality of LOCC.

	Incidence of LOCC	Incidence of LOCC in Males	Incidence of LOCC in Females
Average Level of Tobacco Smoking	*r* = 0.394 *p* < 0.001		
Prevalence of Tobacco Smoking in Males		*r* = 0.286 *p* < 0.001	
Prevalence of Tobacco Smoking in Females			*r* = 0.253 *p* = 0.001
	Mortality of LOCC	Mortality from LOCC in Males	Mortality from LOCC in Females
Average Level of Tobacco Smoking	*r* = 0.214 *p* = 0.005		
Prevalence of Tobacco Smoking in Males		*r* = 0.249 *p* = 0.001	
Prevalence of Tobacco Smoking in Females			*r* = −0.227 *p* = 0.003
	Mortality of LOCC	Mortality from LOCC in Males	Mortality from LOCC in Females
Average Level of Tobacco Chewing	*r* = 0.166 *p* = 0.030		
Prevalence of Tobacco Chewing in Males		----- -----	
Prevalence of Tobacco Chewing in Females			*r* = 0.322 *p* < 0.001

**Table 4:** Correlation of LOCC incidence and mortality with the HDI.

	Incidence of LOCC	Incidence of LOCC in Males	Incidence of LOCC in Females
HDI	*r* = 0.379 *p* < 0.001	*r* = 0.374 *p* < 0.001	*r* = 0.268 *p* < 0.001
Life expectancy	*r* = 0.290 *p* < 0.001	*r* = 0.273 *p* < 0.001	*r* = 0.232 *p* = 0.002
Expected years of schooling	*r* = 0.352 *p* < 0.001	*r* = 0.343 *p* < 0.001	*r* = 0.245 *p* = 0.001
Average years of schooling	*r* = 0.387 *p* < 0.001	*r* = 0.417 *p* < 0.001	*r* = 0.203 *p* = 0.008
	Mortality of LOCC	Mortality from LOCC in Males	Mortality from LOCC in Females
HDI	NS	NS	*r* = −0.319 *p* < 0.001
Life expectancy	*r* = −0.213 *p* = 0.005	NS	*r* = −0.356 *p* < 0.001
Expected years of schooling	NS	NS	*r* = −0.423 *p* < 0.001
Average years of schooling	NS	NS	*r* = −0.307 *p* < 0.001

Notes: NS: non-significant correlations.

## Data Availability

The datasets generated during and/or analyzed during the current study are available from the corresponding author upon reasonable request.

## Supplementary Materials

The following supporting information can be downloaded at: https://www.mdpi.com/article/10.3390/healthcare11081063/s1, Table S1. Lip and oral cavity cancer incidence; Table S2. Lip and oral cavity cancer mortality.
