# Supplementary material for: Lip and Oral Cavity Cancer Incidence and Mortality Rates Associated with Smoking and Chewing Tobacco Use and the Human Development Index in 172 Countries Worldwide: An Ecological Study 2019–2020
Source: Healthcare (Basel). 2023 Apr 7;11(8):1063. doi: 10.3390/healthcare11081063 (PMC10137392; doi:10.3390/healthcare11081063)
Supplement: Supplementary file 1 [file healthcare-11-01063-s001.zip › Table Suppl 1. Lip and oral cavity cancer Incidence.pdf]

Table S1. Lip and oral cavity cancer Incidence.

Country	Rank	Incidence per 100,000	Average Tobacco Smoking per day	Average Tobacco Chewing %	HDI value	Life Expectancy years	Expected Years of Schooling years	Average Years of Schooling years
**Papua New Guinea**	1	27.1	29.3	5.79	0.555	64.5	10.2	4.7
**Pakistan**	2	13.0	14.23	9.47	0.557	67.3	8.3	5.2
**India**	3	12.5	13.05	18.75	0.645	69.7	12.2	6.5
**Sri Lanka**	4	12.4	15.95	9.36	0.782	77.0	14.1	10.6
**Bangladesh**	5	12.2	23.51	23.68	0.632	72.6	11.6	6.2
**Namibia**	6	8.4	17.05	0.87	0.646	63.7	12.6	7.0
**Australia**	7	8.3	15.4	0.41	0.944	83.4	22	12.7
**Hungary**	8	8.1	30.35	0.21	0.854	76.9	15.2	12.0
**Slovakia**	9	7.9	27.1	0.22	0.860	77.5	14.5	12.7
**Cape Verde**	10	7.7	6.19	1.54	0.665	73.0	12.7	6.3
**Latvia**	11	7.7	34.1	0.27	0.866	75.3	16.2	13.0
**Poland**	12	7.7	28.1	0.32	0.880	78.7	16.3	12.5
**France**	13	6.9	34.1	0.13	0.901	82.7	15.6	11.5
**Romania**	14	6.9	30.95	0.36	0.828	76.1	14.3	11.1
**Cuba**	15	6.7	23.55	0.41	0.783	78.8	14.3	11.8
**Portugal**	16	6.7	27.9	0.14	0.864	82.1	16.5	9.3
**United Kingdom**	17	6.5	19.9	0.15	0.932	81.3	17.5	13.2
**Russia**	18	6.5	30.55	0.34	0.824	72.6	15.0	12.2
**Serbia**	19	6.3	38.3	0.45	0.806	76.0	14.7	11.2
**Belgium**	20	5.9	23.15	0.14	0.931	81.6	19.8	12.1
**Afghanistan**	21	5.8	9.83	5.34	0.511	64.8	10.2	3.9
**Spain**	22	5.8	27.65	0.14	0.904	83.6	17.6	10.3
**Montenegro**	23	5.7	35.1	0.53	0.829	76.9	15.0	11.6
**Myanmar**	24	5.7	24.79	10.35	0.583	67.1	10.7	5.0
**Ukraine**	25	5.7	28.2	0.31	0.799	72.1	15.1	11.4
**Republic of Moldova**	26	5.6	23.92	0.25	0.750	71.9	11.5	11.7
**Germany**	27	5.5	26.45	0.18	0.947	81.3	17.0	14.2
**Czech**	28	5.5	28.65	0.39	0.900	79.4	16.8	12.7
**Netherlands**	29	5.5	22.7	0.13	0.944	82.3	18.5	12.4
**Canada**	30	5.4	17.1	0.8	0.929	82.4	16.2	13.4
**Croatia**	31	5.4	35.85	0.31	0.851	78.5	15.2	11.4

**United States of America**	32	5.4	17.6	2.23	0.926	78.9	16.3	13.4
**Luxembourg**	33	5.4	23.75	0.14	0.916	82.3	14.3	12.3
**St. Lucia**	34	5.4	12.23	0.49	0.759	76.2	14.0	8.5
**Belarus**	35	5.3	36.9	0.25	0.823	74.8	15.4	12.3
**World**	36	5.2	19.66	4.71	0.737	72.8	12.7	8.5
**Ireland**	37	5.1	22.35	0.14	0.955	82.3	18.7	12.9
**Thailand**	38	5.1	21.64	1.77	0.777	77.2	15.0	7.9
**Cambodia**	39	5	22.84	7.27	0.594	69.8	11.5	5.0
**Switzerland**	40	5	26.35	0.21	0.955	83.8	16.3	13.4
**Slovenia**	41	4.9	27.35	0.33	0.917	81.3	17.6	12.7
**South Africa**	42	4.9	22.9	1.42	0.709	64.1	13.8	10.2
**New Zealand**	43	4.8	16.8	0.49	0.931	82.3	18.8	12.8
**Laos**	44	4.8	28.04	3.73	0.613	67.9	11.0	5.3
**Lithuania**	45	4.7	29.1	0.22	0.882	75.9	16.6	13.1
**Brazil**	46	4.6	8.88	0.39	0.765	75.9	15.4	8.0
**Denmark**	47	4.6	22.65	0.22	0.940	80.9	18.9	12.6
**Gabon**	48	4.5	12.72	0.39	0.703	66.5	13.0	8.7
**Norway**	49	4.5	18.35	0.82	0.957	82.4	18.1	12.9
**Botswana**	50	4.4	22.34	3.71	0.735	69.6	12.8	9.6
**Austria**	51	4.3	31.1	0.21	0.922	81.5	16.1	12.5
**Japan**	52	4.3	21.8	0.56	0.919	84.6	15.2	12.9
**Nepal**	53	4.3	22.5	23.68	0.602	70.8	12.8	5.0
**Uzbekistan**	54	4.3	13.37	4.39	0.720	71.7	12.1	11.8
**Angola**	55	4.2	10.72	0.46	0.581	61.2	11.8	5.2
**Bulgaria**	56	4.2	37.5	0.68	0.816	75.1	14.4	11.4
**Turkmenistan**	57	4.1	12.32	0.27	0.715	68.2	11.2	10.3
**Bosnia and Herzegovina**	58	3.9	37.8	0.49	0.780	77.4	13.8	9.8
**Estonia**	59	3.9	25.3	0.46	0.892	78.8	16.0	13.1
**Mauricio**	60	3.9	23.3	2.32	0.804	75.0	15.1	9.5
**Samoa**	61	3.9	26.95	1.04	0.715	73.3	12.7	10.8
**Sweden**	62	3.9	13.4	1.05	0.945	82.8	19.5	12.5
**Bhutan**	63	3.8	9.8	20.68	0.654	71.8	13.0	4.1
**Finland**	64	3.8	21.45	0.25	0.938	81.9	19.4	12.8
**Fiji**	65	3.7	28.5	2.53	0.743	67.4	14.4	10.9
**Italy**	66	3.6	23.1	0.15	0.892	83.5	16.1	10.4
**Micronesia**	67	3.6	49.3	7.24	0.620	67.9	11.5	7.8
**Kenya**	68	3.5	10.86	1.28	0.601	66.7	11.3	6.6

**Georgia**	69	3.4	29.41	0.33	0.812	73.8	15.3	13.1
**Kazakhstan**	70	3.4	25.44	0.54	0.825	73.6	15.6	11.9
**Madagascar**	71	3.3	13.73	11.68	0.528	67.0	10.2	6.1
**Mali**	72	3.3	12.47	1.97	0.434	59.3	7.5	2.4
**Uruguay**	73	3.2	27.05	0.18	0.817	77.9	16.8	8.9
**Vanuatu**	74	3.2	21.95	3.21	0.609	70.5	11.7	7.1
**Greece**	75	3.1	38.15	0.15	0.888	82.2	17.9	10.6
**Solomon Islands**	76	3	35.75	2.73	0.567	73.0	10.2	5.7
**Argentina**	77	2.9	24.4	0.2	0.845	76.7	17.7	10.9
**Equatorial Guinea**	78	2.9	15	0.67	0.592	58.7	9.7	5.9
**Singapore**	79	2.9	13.71	0.56	0.938	83.6	16.4	11.6
**Malaysia**	80	2.8	21.75	2.27	0.810	76.2	13.7	10.4
**Qatar**	81	2.8	12.43	0.65	0.848	80.2	12.0	9.7
**Turkey**	82	2.7	30.8	0.36	0.820	77.7	16.6	8.1
**Uganda**	83	2.7	9.39	0.79	0.544	63.4	11.4	6.2
**Indonesia**	84	2.6	30.95	1.22	0.718	71.7	13.6	8.2
**Sudan**	85	2.6	10.72	2.97	0.510	65.3	7.9	3.8
**Mauritania**	86	2.5	18.69	1.01	0.546	64.9	8.6	4.7
**Republic of Korea**	87	2.5	23.85	0.47	0.916	83.0	16.5	12.2
**Albania**	88	2.4	31.7	0.8	0.795	78.6	14.7	10.1
**Eritrea**	89	2.4	6.598	1.57	0.459	66.3	5.0	3.9
**Oman**	90	2.4	8.84	0.71	0.813	77.9	14.2	9.7
**Bolivarian Republic of Venezuela**	91	2.4	17.25	1.32	0.711	72.1	12.8	10.3
**Senegal**	92	2.4	7.83	0.43	0.512	67.9	8.6	3.2
**South Sudan**	93	2.4	11.65	1.21	0.433	57.9	5.3	4.8
**Viet Nam**	94	2.4	24.89	1.03	0.704	75.4	12.7	8.3
**Azerbaijan**	95	2.3	23.05	0.24	0.756	73.0	12.9	10.6
**Cyprus**	96	2.3	33.05	0.15	0.887	81.0	15.2	12.2
**Ethiopia**	97	2.3	5.91	1.26	0.485	66.6	8.8	2.9
**Iceland**	98	2.3	15.45	0.64	0.949	83.0	19.1	12.8
**Morocco**	99	2.3	11.78	1.06	0.686	76.7	13.7	5.6
**Suriname**	100	2.3	21.52	0.58	0.738	71.7	13.2	9.3
**Djibouti**	101	2.3	23.86	1.34	0.524	67.1	6.8	4.1
**Saudi Arabia**	102	2.2	12.38	0.65	0.854	75.1	16.1	10.2
**Kyrgyzstan**	103	2.2	24.12	3.52	0.697	71.5	13.0	11.1

**Lesotho**	104	2.2	21.67	0.99	0.527	54.3	11.3	6.5
**Peru**	105	2.2	5.67	0.6	0.777	76.7	15.0	9.7
**Burkina Faso**	106	2.1	9.23	3.03	0.452	61.6	9.3	1.6
**Burundi**	107	2.1	10.26	2.51	0.433	61.6	11.1	3.3
**Philippines**	108	2.1	24.57	1.69	0.718	71.2	13.1	9.4
**Israel**	109	2.1	20.65	0.19	0.919	83.0	16.2	13.0
**Kuwait**	110	2.1	19.21	0.91	0.806	75.5	14.2	7.3
**Malawi**	111	2.1	13.55	0.84	0.483	64.3	11.2	4.7
**Malta**	112	2.1	24.45	0.15	0.895	82.5	16.1	11.3
**Niger**	113	2.1	7.86	2.17	0.394	62.4	6.5	2.1
**Trinidad and Tobago**	114	2.1	18.53	0.43	0.796	73.5	13.0	11.0
**Tunisia**	115	2.1	24.05	2.41	0.740	76.7	15.1	7.2
**Bahrain**	116	2	14.25	1.03	0.852	77.3	16.3	9.5
**Chad**	117	2	9.41	1.33	0.398	54.2	7.3	2.5
**Egypt**	118	2	22.23	0.48	0.707	72.0	13.3	7.4
**Tanzania**	119	2	10.48	0.77	0.529	65.5	8.1	6.1
**Cameroon**	120	1.9	8.15	0.92	0.563	59.3	12.1	6.3
**United Arab Emirates**	121	1.9	10.84	1.1	0.890	78.0	14.3	12.1
**Mozambique**	122	1.9	14.77	1.78	0.456	60.9	10.0	3.5
**Paraguay**	123	1.9	16.46	1.3	0.728	74.3	12.7	8.5
**Barbados**	124	1.8	9.25	0.34	0.814	79.2	15.4	10.6
**Colombia**	125	1.8	12.21	0.58	0.767	77.3	14.4	8.5
**Haiti**	126	1.8	6.37	0.41	0.510	64.0	9.7	5.6
**Mongolia**	127	1.8	29.98	1.19	0.737	69.9	14.2	10.3
**Central African Republic**	128	1.8	8.3	0.67	0.397	53.3	7.6	4.3
**Togo**	129	1.8	8.13	0.87	0.515	61.0	12.7	4.9
**China**	130	1.7	26.62	0.54	0.761	76.9	14.0	8.1
**El Salvador**	131	1.7	10.5	0.52	0.673	73.3	11.7	6.9
**Guyana**	132	1.7	14.55	0.42	0.682	69.9	11.4	8.5
**Jordan**	133	1.7	32.4	1.11	0.729	74.5	11.4	10.5
**North Macedonia**	134	1.7	39.05	0.44	0.774	75.8	13.6	9.8
**Islamic Republic of Iran**	135	1.7	14.81	0.89	0.783	76.7	14.8	10.3
**Tajikistan**	136	1.7	9.09	3.31	0.668	71.1	11.7	10.7
**Côte d'Ivoire**	137	1.6	12.79	0.62	0.538	57.8	10.0	5.3
**Guatemala**	138	1.6	12.42	0.28	0.663	74.3	10.8	6.6

**Guinea-Bissau**	139	1.6	4.76	0.83	0.480	58.3	10.6	3.6
**Liberia**	140	1.6	8.13	0.66	0.480	64.1	9.6	4.8
**Maldives**	141	1.6	26.92	4.62	0.740	78.9	12.2	7.0
**Rwanda**	142	1.6	15.11	1.36	0.543	69.0	11.2	4.4
**Costa Rica**	143	1.5	11.24	0.22	0.810	80.3	15.7	8.7
**Ecuador**	144	1.5	14.83	0.21	0.759	77.0	14.6	8.9
**Nigeria**	145	1.5	4.29	0.39	0.539	54.7	10.0	6.7
**Sierra Leone**	146	1.5	19.17	1.26	0.452	54.7	10.2	3.7
**Syria**	147	1.5	24.05	0.4	0.567	72.7	8.9	5.1
**Timor-Leste**	148	1.5	34.87	1.82	0.606	69.5	12.6	4.8
**Honduras**	149	1.4	14.62	0.37	0.634	75.3	10.1	6.6
**Iraq**	150	1.4	20.56	0.41	0.674	70.6	11.3	7.3
**Lebanon**	151	1.4	36.05	0.87	0.744	78.9	11.3	8.7
**Libya**	152	1.4	20.03	0.83	0.724	72.9	12.9	7.6
**Mexico**	153	1.4	18.01	0.39	0.779	75.1	14.8	8.8
**Dominican Republic**	154	1.4	11.48	0.86	0.756	74.1	14.2	8.1
**Armenia**	155	1.3	29.2	0.22	0.776	75.1	13.1	11.3
**Bahamas**	156	1.3	7.29	0.37	0.814	73.9	12.9	11.4
**Comoros**	157	1.3	13.14	5.4	0.554	64.3	11.2	5.1
**Eswatini**	158	1.3	8.93	0.38	0.611	60.2	11.8	6.9
**Democratic Republic of Congo**	159	1.3	11.48	1.15	0.480	60.7	9.7	6.8
**Benin**	160	1.2	6.14	1.88	0.545	61.8	12.6	3.8
**Chile**	161	1.2	34.45	0.21	0.851	80.2	16.4	10.6
**Ghana**	162	1.2	6.29	0.81	0.611	64.1	11.5	7.3
**Guinea**	163	1.2	15.61	0.83	0.477	61.6	9.4	2.8
**Panama**	164	1.2	8.48	0.37	0.815	78.5	12.9	10.2
**Jamaica**	165	0.96	13.46	0.46	0.734	74.5	13.1	9.7
**Algeria**	166	0.86	17.22	4.01	0.748	76.9	14.6	8.0
**Bolivia**	167	0.79	12.75	0.75	0.718	71.5	14.2	9.0
**Brunei Darussalam**	168	0.75	16.94	0.87	0.838	75.9	14.3	9.1
**Congo**	169	0.67	11.25	0.66	0.574	64.6	11.7	6.5
**Nicaragua**	170	0.64	13.48	0.65	0.660	74.5	12.3	6.9
**Belize**	171	0	13.31	0.52	0.716	74.6	13.1	9.9
**São Tomé and Príncipe**	172	0	4.75	0.47	0.625	70.4	12.7	6.4
