# Supplementary material for: Lip and Oral Cavity Cancer Incidence and Mortality Rates Associated with Smoking and Chewing Tobacco Use and the Human Development Index in 172 Countries Worldwide: An Ecological Study 2019–2020
Source: Healthcare (Basel). 2023 Apr 7;11(8):1063. doi: 10.3390/healthcare11081063 (PMC10137392; doi:10.3390/healthcare11081063)
Supplement: Supplementary file 1 [file healthcare-11-01063-s001.zip › Table Suppl 2. Lip and oral cavity cancer mortality.pdf]

Table S2. Lip and oral cavity cancer mortality.

Country	Rank	Mortality per 100,000	Average Tobacco Smoking per day	Average Tobacco Chewing %	HDI value	Life Expectancy years	Expected Years of Schooling years	Average Years of Schooling years
**Papua New Guinea**	1	10.6	29.3	5.79	0.555	64.5	10.2	4.7
**Pakistan**	2	8.2	14.23	9.47	0.557	67.3	8.3	5.2
**Bangladesh**	3	7.2	23.51	23.68	0.632	72.6	11.6	6.2
**India**	4	7.0	13.05	18.75	0.645	69.7	12.2	6.5
**Sri Lanka**	5	5.8	15.95	9.36	0.782	77.0	14.1	10.6
**Namibia**	6	4.7	17.05	0.87	0.646	63.7	12.6	7.0
**Afghanistan**	7	4.2	9.83	5.34	0.511	64.8	10.2	3.9
**Hungary**	8	4.1	30.35	0.21	0.854	76.9	15.2	12.0
**Belarus**	9	4.0	36.9	0.25	0.823	74.8	15.4	12.3
**Polond**	10	3.6	28.1	0.32	0.880	78.7	16.3	12.5
**Lithuania**	11	3.5	29.1	0.22	0.882	75.9	16.6	13.1
**Myanmar**	12	3.4	24.79	10.35	0.583	67.1	10.7	5.0
**Republic of Moldova**	13	3.4	23.92	0.25	0.750	71.9	11.5	11.7
**Slovakia**	14	3.3	27.1	0.22	0.860	77.5	14.5	12.7
**Romania**	15	3.3	30.95	0.36	0.828	76.1	14.3	11.1
**Cambodia**	16	3.2	22.84	7.27	0.594	69.8	11.5	5.0
**Latvia**	17	3.2	34.1	0.27	0.866	75.3	16.2	13.0
**Montenegro**	18	3.2	35.1	0.53	0.829	76.9	15.0	11.6
**Ukraine**	19	3.1	28.2	0.31	0.779	72.1	15.1	11.4
**Russia**	20	3.0	30.55	0.34	0.824	72.6	15.0	12.2
**Cape Verde**	21	2.9	6.19	1.54	0.665	73.0	12.7	6.3
**Laos**	22	2.8	28.04	3.73	0.613	67.9	11.0	5.3
**Serbia**	23	2.7	38.3	0.45	0.806	76.0	14.7	11.2
**Angola**	24	2.6	10.72	0.46	0.581	61.2	11.8	5.2
**Bhutan**	25	2.6	9.8	20.68	0.654	71.8	13.0	4.1
**Mali**	26	2.5	12.47	1.97	0.434	59.3	7.5	2.4
**Thailand**	27	2.5	21.64	1.77	0.777	77.2	15.0	7.9
**Turkmenistan**	28	2.4	12.32	0.27	0.715	68.2	11.2	10.3
**World**	29	2.4	19.66	4.71	0.737	72.8	12.7	8.5
**Cuba**	30	2.3	23.55	0.41	0.783	78.8	14.3	11.8
**Gabon**	31	2.3	12.72	0.39	0.703	66.5	13.0	8.7

**Nepal**	32	2.3	22.5	23.68	0.602	70.8	12.8	5.0
**Uzbekistan**	33	2.3	13.37	4.39	0.720	71.7	12.1	11.8
**Kazakhstan**	34	2.2	25.44	0.54	0.825	73.6	15.6	11.9
**Kenya**	35	2.2	10.86	1.28	0.601	66.7	11.3	6.6
**Madagascar**	36	2.2	13.73	11.68	0.528	67.0	10.2	6.1
**Portugal**	37	2.2	27.9	0.14	0.864	82.1	16.5	9.3
**Czech**	38	2.1	28.65	0.39	0.900	79.4	16.8	12.7
**South Africa**	39	2.1	22.9	1.42	0.709	64.1	13.8	10.2
**Botswana**	40	2.0	22.34	3.71	0.735	69.6	12.8	9.6
**Croatia**	41	2.0	35.85	0.31	0.851	78.5	15.2	11.4
**Estonia**	42	2.0	25.3	0.46	0.892	78.8	16.0	13.1
**Equatorial Guinea**	43	2.0	15.0	0.67	0.592	58.7	9.7	5.9
**Solomon Islands**	44	2.0	35.75	2.73	0.567	73.0	10.2	5.7
**Mauricio**	45	2.0	23.3	2.32	0.804	75.0	15.1	9.5
**Suriname**	46	2.0	21.52	0.58	0.738	71.7	13.2	9.3
**Austria**	47	1.9	31.1	0.21	0.922	81.5	16.1	12.5
**Brazil**	48	1.9	8.88	0.39	0.765	75.9	15.4	8.0
**Mauritania**	49	1.9	18.69	1.01	0.546	64.9	8.6	4.7
**Uganda**	50	1.9	9.39	0.79	0.544	63.4	11.4	6.2
**Uruguay**	51	1.8	27.05	0.18	0.817	77.9	16.8	8.9
**Djibouti**	52	1.8	23.86	1.34	0.524	67.1	6.8	4.1
**Eritrea**	53	1.7	6.598	1.57	0.459	66.3	5.0	3.9
**Fiji**	54	1.7	28.5	2.53	0.743	67.4	14.4	10.9
**Georgia**	55	1.7	29.41	0.33	0.812	73.8	15.3	13.1
**South Sudan**	56	1.7	11.65	1.21	0.433	57.9	5.3	4.8
**Belgium**	57	1.6	23.15	0.14	0.931	81.6	19.8	12.1
**Borundi**	58	1.6	10.26	2.51	0.433	61.6	11.1	3.3
**Chad**	59	1.6	9.41	1.33	0.398	54.2	7.3	2.5
**Ethiopía**	60	1.6	5.91	1.26	0.485	66.6	8.8	2.9
**Niger**	61	1.6	7.86	2.17	0.394	62.4	6.5	2.1
**Qatar**	62	1.6	12.43	0.65	0.848	80.2	12.0	9.7
**Samoa**	63	1.6	26.95	1.04	0.715	73.3	12.7	10.8
**Senegal**	64	1.6	7.83	0.43	0.512	67.9	8.6	3.2
**Bosnia and Herzegovina**	65	1.5	37.8	0.49	0.780	77.4	13.8	9.8
**Bulgaria**	66	1.5	37.5	0.68	0.816	75.1	14.4	11.4
**Burkina Faso**	67	1.5	9.23	3.03	0.452	61.6	9.3	1.6

**France**	68	1.5	34.1	0.13	0.901	82.7	15.6	11.5
**Malaysia**	69	1.5	21.75	2.27	0.810	76.2	13.7	10.4
**Malawi**	70	1.5	13.55	0.84	0.483	64.3	11.2	4.7
**Micronesia**	71	1.5	49.3	7.24	0.620	67.9	11.5	7.8
**United Kingdom**	72	1.5	19.9	0.15	0.932	81.3	17.5	13.2
**St. Lucia**	73	1.5	12.23	0.49	0.759	76.2	14.0	8.5
**Germany**	74	1.4	26.45	0.18	0.947	81.3	17.0	14.2
**Denmark**	75	1.4	22.65	0.22	0.940	80.9	18.9	12.6
**Spain**	76	1.4	27.65	0.14	0.904	83.6	17.6	10.3
**Indonesia**	77	1.4	30.95	1.22	0.718	71.7	13.6	8.2
**Kyrgyzstan**	78	1.4	24.12	3.52	0.697	71.5	13.0	11.1
**Lesotho**	79	1.4	21.67	0.99	0.527	54.3	11.3	6.5
**Malta**	80	1.4	24.45	0.15	0.895	82.5	16.1	11.3
**Mozambique**	81	1.4	14.77	1.78	0.456	60.9	10.0	3.5
**Central African Republic**	82	1.4	8.3	0.67	0.397	53.3	7.6	4.3
**Switzerland**	83	1.4	26.35	0.21	0.955	83.8	16.3	13.4
**Cameroon**	84	1.3	8.15	0.92	0.563	59.3	12.1	6.3
**Finland**	85	1.3	21.45	0.25	0.938	81.9	19.4	12.8
**Ireland**	86	1.3	22.35	0.14	0.955	82.3	18.7	12.9
**Morocco**	87	1.3	11.78	1.06	0.686	76.7	13.7	5.6
**Sudan**	88	1.3	10.72	2.97	0.510	65.3	7.9	3.8
**Tanzania**	89	1.3	10.48	0.77	0.529	65.5	8.1	6.1
**Timor-Leste**	90	1.3	34.87	1.82	0.606	69.5	12.6	4.8
**Philipines**	91	1.2	24.57	1.69	0.718	71.2	13.1	9.4
**Guinea-Bissau**	92	1.2	4.76	0.83	0.480	58.3	10.6	3.6
**Italy**	93	1.2	23.1	0.15	0.892	83.5	16.1	10.4
**Mongolia**	94	1.2	29.98	1.19	0.737	69.9	14.2	10.3
**Sierra Leone**	95	1.2	19.17	1.26	0.452	54.7	10.2	3.7
**Togo**	96	1.2	8.13	0.87	0.515	61.0	12.7	4.9
**Viet Nam**	97	1.2	24.89	1.03	0.704	75.4	12.7	8.3
**Albanian**	98	1.1	31.7	0.8	0.795	78.6	14.7	10.1
**Argentina**	99	1.1	24.4	0.2	0.845	76.7	17.7	10.9
**Azerbaijan**	100	1.1	23.05	0.24	0.756	73.0	12.9	10.6
**Bahamas**	101	1.1	7.29	0.37	0.814	73.9	12.9	11.4
**Barbados**	102	1.1	9.25	0.34	0.814	79.2	15.4	10.6
**Canada**	103	1.1	17.7	0.8	0.929	82.4	16.2	13.4

**Côte d'Ivoire**	104	1.1	12.79	0.62	0.538	57.8	10.0	5.3
**Slovenia**	105	1.1	27.35	0.33	0.917	81.3	17.6	12.7
**Iceland**	106	1.1	15.45	0.64	0.949	83.0	19.1	12.8
**Liberia**	107	1.1	8.13	0.66	0.480	64.1	9.6	4.8
**New Zealand**	108	1.1	16.8	0.49	0.931	82.3	18.8	12.8
**Oman**	109	1.1	8.84	0.71	0.813	77.9	14.2	9.7
**Rwanda**	110	1.1	15.11	1.36	0.543	69.0	11.2	4.4
**Syria**	111	1.1	24.05	0.4	0.567	72.7	8.9	5.1
**Sweden**	112	1.1	13.4	1.05	0.945	82.8	19.5	12.5
**Tajikistan**	113	1.1	9.09	3.31	0.668	71.1	11.7	10.7
**Trinidad and Tobago**	114	1.1	18.53	0.43	0.796	73.5	13.0	11.0
**El Salvador**	115	1.0	10.5	0.52	0.673	73.3	11.7	6.9
**Greece**	116	1.0	38.15	0.15	0.888	82.2	17.9	10.6
**Guyana**	117	1.0	14.55	0.42	0.682	69.9	11.4	8.5
**Netherlands**	118	1.0	22.7	0.13	0.944	82.3	18.5	12.4
**Nigeria**	119	1.0	4.29	0.39	0.539	54.7	10.0	6.7
**Bolivarian Republic of Venezuela**	120	1.0	17.25	1.32	0.711	72.1	12.8	10.3
**Singapore**	121	1.0	13.71	0.56	0.938	83.6	16.4	11.6
**Saudi Arabia**	122	0.97	12.38	0.65	0.854	75.1	16.1	10.2
**Tunisia**	123	0.97	24.05	2.41	0.740	76.7	15.1	7.2
**Australia**	124	0.94	15.4	0.41	0.944	83.4	22.0	12.7
**Democratic Republic of Congo**	125	0.94	11.48	1.15	0.480	60.7	9.7	6.8
**Kuwait**	126	0.93	19.21	0.91	0.806	75.5	14.2	7.3
**Norway**	127	0.93	18.35	0.82	0.957	82.4	18.1	12.9
**Japan**	128	0.92	21.8	0.56	0.919	84.6	15.2	12.9
**North Macedonia**	129	0.92	39.05	0.44	0.774	75.8	13.6	9.8
**Guinea**	130	0.89	15.61	0.83	0.477	61.6	9.4	2.8
**Haiti**	131	0.88	6.37	0.41	0.510	64.0	9.7	5.6
**Egypt**	132	0.87	22.23	0.48	0.707	72.0	13.3	7.4
**Jordan**	133	0.86	32.4	1.11	0.729	74.5	11.4	10.5
**Paraguay**	134	0.86	16.46	1.3	0.728	74.3	12.7	8.5
**Comoros**	135	0.84	13.14	5.4	0.554	64.3	11.2	5.1
**United States Of America**	136	0.83	17.6	2.23	0.926	78.9	16.3	13.4
**Barhain**	137	0.81	14.25	1.03	0.852	77.3	16.3	9.5
**China**	138	0.81	26.62	0.54	0.761	76.9	14.0	8.1

**United Arab Emirates**	139	0.81	10.84	1.1	0.890	78.0	14.3	12.1
**Eswatini**	140	0.81	8.93	0.38	0.611	60.2	11.8	6.9
**Iraq**	141	0.81	20.56	0.41	0.674	70.6	11.3	7.3
**Vanuatu**	142	0.81	21.95	3.21	0.609	70.5	11.7	7.1
**Benin**	143	0.80	6.14	1.88	0.545	61.8	12.6	3.8
**Peru**	144	0.80	5.67	0.6	0.777	76.7	15.0	9.7
**Guatemala**	145	0.78	12.42	0.28	0.663	74.3	10.8	6.6
**Luxembourg**	146	0.75	23.75	0.14	0.916	82.3	14.3	12.3
**Ghana**	147	0.74	6.29	0.81	0.611	64.1	11.5	7.3
**Colombia**	148	0.72	12.21	0.58	0.767	77.3	14.4	8.5
**Armenia**	149	0.71	29.2	0.22	0.776	75.1	13.1	11.3
**Cyprus**	150	0.71	33.05	0.15	0.887	81.0	15.2	12.2
**Turkey**	151	0.71	30.8	0.36	0.820	77.7	16.6	8.1
**Libya**	152	0.70	20.03	0.83	0.724	72.9	12.9	7.6
**Panama**	153	0.68	8.48	0.37	0.815	78.5	12.9	10.2
**Republic of Korea**	154	0.68	23.85	0.47	0.916	83.0	16.5	12.2
**Islamic Republic of Iran**	155	0.68	14.81	0.89	0.783	76.7	14.8	10.3
**Lebanon**	156	0.67	36.05	0.87	0.744	78.9	11.3	8.7
**Ecuador**	157	0.65	14.83	0.21	0.759	77.0	14.6	8.9
**Costa Rica**	158	0.64	11.24	0.22	0.810	80.3	15.7	8.7
**Chile**	159	0.52	34.45	0.21	0.851	80.2	16.4	10.6
**Mexico**	160	0.52	18.01	0.39	0.779	75.1	14.8	8.8
**Dominican Republic**	161	0.52	11.48	0.86	0.756	74.1	14.2	8.1
**Honduras**	162	0.48	14.62	0.37	0.634	75.3	10.1	6.6
**Israel**	163	0.47	20.65	0.19	0.919	83.0	16.2	13.0
**Jamaica**	164	0.46	13.46	0.46	0.734	74.5	13.1	9.7
**Congo**	165	0.45	11.25	0.66	0.574	64.6	11.7	6.5
**Nicaragua**	166	0.43	13.48	0.65	0.660	74.5	12.3	6.9
**Brunei Darussalam**	167	0.41	16.94	0.87	0.838	75.9	14.3	9.1
**Bolivia**	168	0.38	12.75	0.75	0.718	71.5	14.2	9.0
**Algeria**	169	0.37	17.22	4.01	0.748	76.9	14.6	8.0
**Belize**	170	0	13.31	0.52	0.716	74.6	13.1	9.9
**Maldives**	171	0	26.92	4.62	0.740	78.9	12.2	7.0
**São Tomé and Príncipe**	172	0	4.75	0.47	0.625	70.4	12.7	6.4
